# 

**Published:** 1899-01-07

**Authors:** 


**Notices of Births, Deaths, and Marriages** are inserted in this column at a charge of 2s. 6d. for an announcement not exceeding four lines.

### NOTICE TO CORRESPONDENTS.

All MS., Letters, Books for Review, and other matters intended for the Editor should be addressed THE EDITOR, "THE HOSPITAL," THE HOSPITAL BUILDING, 28 & 29, SOUTHAMPTON STREET, STRAND, W.C.

The Editor cannot undertake to return rejected MS., even when accompanied by stamped directed envelope.

**Notice to Advertisers and Subscribers.**

All Advertisements, Orders for Copies of THE HOSPITAL, and Business Communications should be addressed to **THE MANAGER (Not to the Editor),** THE HOSPITAL, The Hospital Building, 28 & 29, Southampton Street, Strand, London, W.C.

The Cost of Subscription, post free, is as follows:—Per week, 2½d.; per quarter, 2s. 8d.; per half-year, 5s. 3d.; per annum, 10s. 6d. Foreign:—Per annum, 15s. 2d., payable, in all cases, in advance.

**NOTICE.—Vol. XXIV. of "The Hospital" (April, 1898, to September, 1898), in handsome cloth binding, is now on sale at the Office of this Journal, price 7s. 6d. Cases for binding the Numbers contained in this Volume 1s. 6d. Index to Vol. XXIV., 2½d., post free.**

**The Monthly Part for January is now ready. Price 6d., by post 8d.**

### Scraps and Gleanings.

The Queen has forwarded a gift of twenty pheasants for the patients of the Cancer Hospital, Brompton.

The Wigan Infirmary Board have before them a scheme for providing an isolation block separate from the hospital proper, the erection of which, according to the Special Committee appointed by the Board to consider the question and take architect's advice, is an urgent necessity. The difficulty in the way is the lack of funds.

The Public Health Committee for Forfar have before them a proposal that the Local Authority should acquire from the Dundee and Forfar District Committee of the County Council a right to the use of the Fever Hospital to be erected by the Committee, the accommodation at the disposal of the Forfar Infirmary being quite insufficient in the event of an outbreak of infectious disease.

### The Student of Medicine.

### APPOINTMENTS.

V. Burrows, M.B., B.S.Durh., M.R.C.S., appointed Medical Officer for the No. 2 District of the Aylesbury Union. T. W. Carlow, M.D.(Canada), appointed Clinical Assistant to the Chelsea Hospital for Women. Dr. Cotes, appointed Medical Officer for the Great Barford District of the Bedford Union. A. A. G. Dickey, M.D.R.U.I., J.P., appointed Medical Officer and Public Vaccinator for the Colne District of the Burnley Union. W. C. Hamilton, M.B., C.M., appointed Certifying Factory Surgeon for Plymouth. G. B. Hillman, L.S.A., appointed Medical Officer of Health to the Castleford Union Council. R. G. McKerron, M.A., M.D., appointed Senior Physician to the Royal Hospital for Sick Children, Aberdeen. D. Orr, M.B., appointed Pathologist to the County Asylum, Prestwich, Manchester. M. J. Ross, M.B., appointed Resident Medical Officer for the Workhouse of the Newcastle Urban. F. W. Sumner, B.A.Cantab., M.R.C.S., L.R.C.P., appointed House Physician to St. Mary's Hospital, London.

